# Natural Deep Eutectic Solvents for the Extraction of Spilanthol from *Acmella oleracea* (L.) R.K.Jansen

**DOI:** 10.3390/molecules29030612

**Published:** 2024-01-27

**Authors:** Fabian Alperth, Theresa Feistritzer, Melanie Huber, Olaf Kunert, Franz Bucar

**Affiliations:** 1 Institute of Pharmaceutical Sciences, Department of Pharmacognosy, University of Graz, Beethovenstraße 8, 8010 Graz, Austria; fabian.alperth@uni-graz.at (F.A.);; 2 Institute of Pharmaceutical Sciences, Department of Pharmaceutical Chemistry, University of Graz, Universitätsplatz 1, 8010 Graz, Austria; olaf.kunert@uni-graz.at

**Keywords:** *Acmella oleracea*, spilanthol, green extraction, NADES, UHPLC-MS, HPLC-DAD, isolation, NMR

## Abstract

With a growing focus on green chemistry, the extraction of natural products with natural deep eutectic solvents (NADES), which are eutectic mixtures of hydrogen bond donors and acceptors, has become an ever-expanding field of research. However, the use of NADES for the extraction of spilanthol from *Acmella oleracea* (L.) R.K.Jansen has not yet been investigated. Therefore, in this study, 20 choline chloride-based NADES, and for comparison, ethanol, were used as green extraction agents for spilanthol from *Acmella oleracea* flower heads. The effects of time, water addition, and temperature on NADES extractions were investigated and analysed by HPLC-DAD quantification. Additionally, UHPLC-DAD-ESI-MS^n^ results for dichloromethane extracts, as well as the isolation of spilanthol and other main constituents as reference compounds, are reported. The best green extraction results were achieved by choline chloride (ChCl) with 1,2-propanediol (P, 1:2 molar ratio, +20% water) at 244.58 µg/mL, comparable to yields with ethanol (245.93 µg/mL). Methylurea (MeU, 1:2, +20% water) also showed promising results as a hydrogen bond donor in combination with choline chloride (208.12 µg/mL). In further experiments with NADES ChCl/P (1:2) and ChCl/MeU (1:2), extraction time had the least effect on spilanthol extraction with NADES, while yield decreased with water addition over 20% and increased with extraction temperature up to 80 °C. NADES are promising extraction agents for the extraction of spilanthol, and these findings could lead to applicable extracts for medicinal purposes, due to their non-toxic constituents.

## 1. Introduction

*Acmella oleracea* (L.) R.K.Jansen is an herbaceous plant in the Asteraceae family, believed to be cultivated from *A. alba*, native to Peru. The plant is primarily known for cultivation with long-established economic value but can be found in the wild. It has broadly ovate leaves and characteristic discoid flower heads [1]. Apart from ornamental and culinary use for its aesthetic and particular spicy flavour, the plant’s ethnobotanical application is mostly medicinal, with its anaesthetic use against toothache being the most prominent [2]. Major bioactive compounds in *A. oleracea* belong to the group of N-alkylamides, with the primary constituent being spilanthol (2E,6Z,8E-N-isobutyl-2,6,8-decatrienamide) [3,4,5,6]. This substance and alkylamide-rich extracts of *A. oleracea* have found recognition in research for their pharmacological potential. Investigations suggest that the analgesic and antinociceptive effects of spilanthol involve the activation of opioidergic, serotoninergic, and GABAergic systems, with an increased release of GABA mediated by the alkylamide [7,8]. In vivo studies prove the antinociceptive effect in several different mouse pain models [9,10,11]. Additional in silico molecular docking evaluations show that alkylamides from *A. oleracea*, including spilanthol, can interact with CB1, CB2, and TRPV1 receptors [11]. Anti-inflammatory effects have been shown for spilanthol in dermatitis, pancreatitis, and intestinal mucositis models in mice, with the suppression of inflammatory transcription factors and inhibited iNOS expression [12,13]. *A. oleracea* extracts rich in alkylamides also show antimicrobial activity against *Salmonella typhi* and antibiofilm activity against *Streptococcus mutans* biofilms [14,15]. Spilanthol inhibits aflatoxin biosynthesis and the growth of *Aspergillus parasiticus* [16]. Cosmetics is another developing field of application for *A. oleracea* and spilanthol, with extracts showing in vivo improvement of human skin wrinkle parameters after two weeks of application [17].

Deep eutectic solvents (DESs) were first described twenty years ago with eutectic mixtures of quaternary ammonium salts (choline chloride) as hydrogen bond acceptors (HBAs) and a range of amides and carboxylic acids as hydrogen bond donors (HBDs), which are liquid at ambient temperatures. Interesting solvent properties of DES based on hydrogen bonding were recognized early on [18,19]. The known range of HBAs and HBDs to form DES increased rapidly. Also, the possibility of tailoring DES properties depending on the HBD in use became obvious [20]. A similarly famous group of novel solvents is called ionic liquids, molten salts with low melting temperatures. Their use as solvent media has found wide application and developed to the preparation of magnetic ionic liquids, which have been applied successfully for chiral and amino acid separation in aqueous two-phase systems, the recovery of phenolic compounds from aqueous solutions, and the enrichment and detection of trace quinolones in milk [21,22,23,24]. In 2011, the term “natural deep eutectic solvents” (NADES) was introduced for eutectic mixtures that consist of simple molecules present in most living cells. It is hypothesized that they play a crucial part in living cells as solvents for the biosynthesis and storage of natural products, in addition to water and lipid phases [25]. Furthermore, it was proposed that the intestinal mucous layer possesses NADES qualities, building the necessary environment for the solution of substrates and localisation of digestive enzymes, and therefore suggesting the importance of NADES in animal and human physiology [26]. Recent studies suggest that choline chloride-based NADES inhibit collagenase and elastase, and can be applied in stable cosmetic formulations, therefore showing potential for their application in the cosmetics industry [27]. In accordance with these properties and the growing focus on green extraction, non-toxic solvents, low-cost materials, and easy methodologies, the extraction of natural products with different NADES has become an ever-expanding field of research that has already been reviewed in detail [28,29,30]. However, the use of NADES for the extraction of spilanthol from *A. oleracea* has not been investigated. To date, extraction methods for spilanthol in the literature include classical (Soxhlet extraction, heating, stirring, maceration), ultrasound, and microwave-assisted organic solvent extractions [31,32], and supercritical carbon dioxide extraction [33,34,35].

Therefore, the goal of this study is the first comparison between choline chloride-based NADES and ethanol as green extraction agents for spilanthol from *Acmella oleracea* and the investigation of factors of time, water addition, and temperature on NADES extractions. Additionally, the necessary isolation of spilanthol as a reference compound for quantifications and other main constituents, as well as UHPLC-DAD-ESI-MS^n^ results are reported. Findings in spilanthol extraction with NADES could lead to applicable extracts for medicinal purposes, due to non-toxic constituents.

## 2. Results and Discussion

### 2.1. UHPLC-DAD-ESI-MS^n^ Analysis and Isolation

**Figure 1:**
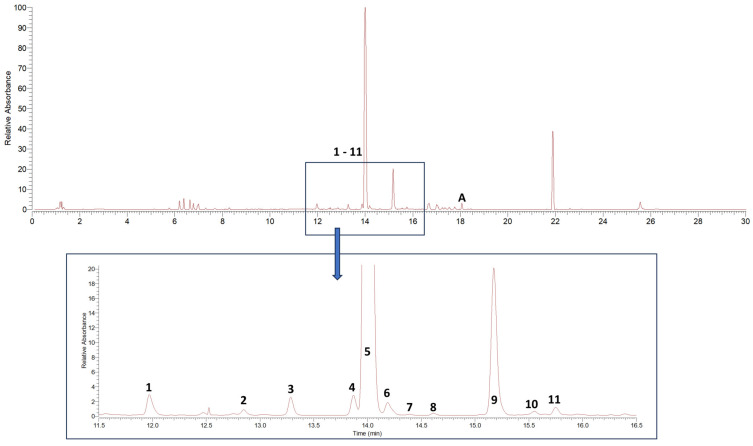
DAD-UV chromatogram (spectrum maximum plot 190–700 nm) of *Acmella oleracea* flower heads dichloromethane extract. **1**–**11** characterized alkylamides, **A** additionally isolated ketoester (acmellonate).

Dichloromethane extracts were used for the initial qualitative analysis of alkylamide composition in the *Acmella oleracea* flower heads utilized in this study and for the subsequent isolation of reference substances. The drug extract ratios (DERs) of two individual extracts, after drying on a rotary evaporator, were determined to be 8.62 to 1 and 8.85 to 1, resulting in extract yields of 11.60% and 11.30%, respectively. Interpretation of MS^n^ data in positive ionization mode showed the presence of 11 known alkylamides. Results are given in Table 1, listing fragment ions in MS^n^ of ≥10% relative intensity. Figure 1 shows the corresponding DAD-UV chromatogram (190–700 nm).

**Figure 2:**
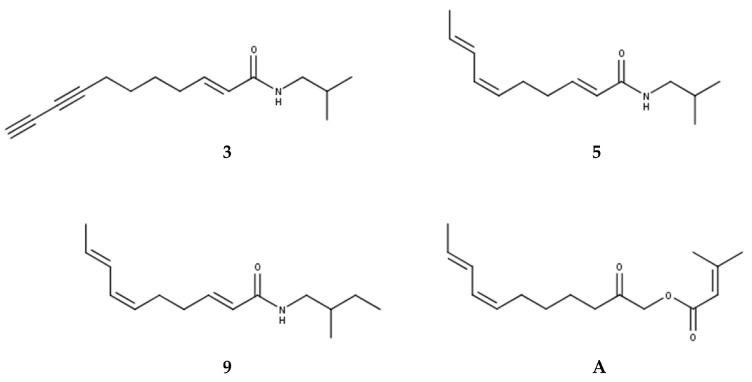
Structures of isolated alkylamides (**3**, **5**, **9**) and ketoester (**A**).

All tentatively identified alkylamides eluted within retention times of about 12 to 16 min. Characteristic fragmentations associated with the amide group, showing losses of 70, 87, 113, or 115 amu, supported the identification of **7** and **9** as methylbutylamides. All others were isobutylamides with characteristic losses of 56, 73, 99 or 101 amu [3,4,36]. Three alkylamides (**3**, **5**, **9**) could be confirmed by NMR and GC-MS (Supplementary Materials Tables S1–S3, Figures S1–S3 and S5–S7) after fractionation of the dichloromethane extract by SPE, followed by isolation using semipreparative HPLC. Additionally, the ketoester acmellonate (substance **A**, Figure 1 and Figure 2), could be isolated and confirmed by NMR (Supplementary Materials Table S4, Figure S4). For this substance, only two previous references of occurrence in *Acmella oleracea* can be found in the literature [31,37]. Spilanthol (**5**) was the most abundant alkylamide and could be obtained with a purity of 97%, as confirmed by NMR. It was used as a reference compound for further investigations. Figure 2 shows the structures of all isolated compounds.

### 2.2. Screening of Spilanthol Extraction with NADES and Ethanol

**Figure 3:**
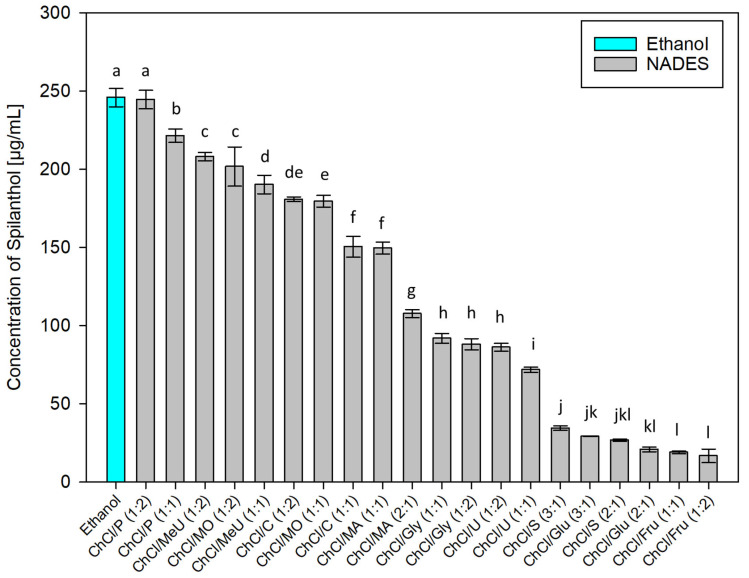
Concentration of spilanthol in extracts prepared with NADES and ethanol; data are given as means ± SD and different letters indicate significant differences between extracts (*p* < 0.05).

**Figure 4:**
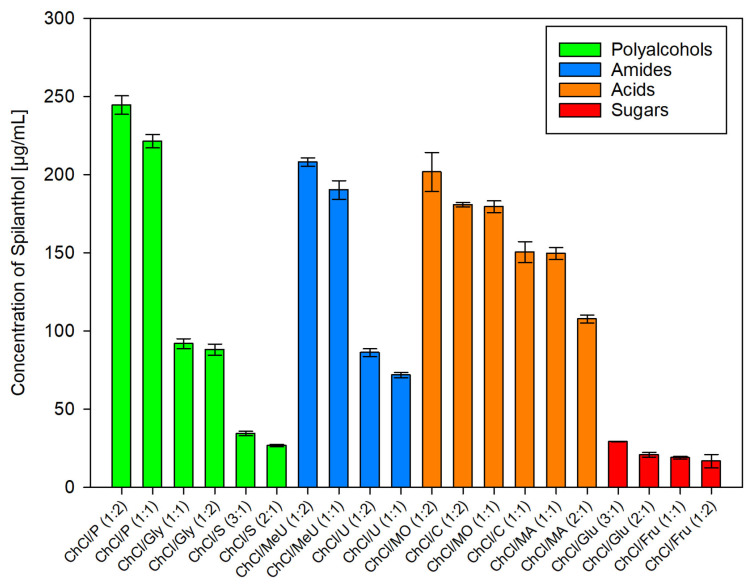
Concentration of spilanthol in NADES extracts grouped by HBD substance class; data are given as means ± SD.

After employing dichloromethane extraction for qualitative analysis and isolation of main compounds in *Acmella oleracea* flower heads, green chemistry alternatives for the extraction of spilanthol were investigated. For this, 20 different NADES preparations and ethanol as reference for a classic green organic solvent, were used as extraction agents. In NADES, choline chloride (ChCl), which was granted GRAS status (generally recognized as safe) by the United States Food and Drug Administration (FDA) [38], was chosen as a non-toxic hydrogen bond acceptor (HBA) in all cases. Hydrogen bond donors (HBD) can be separated into two amides (methylurea MeU, urea U), three polyalcohols (1,2-propanediol P, glycerol Gly, sorbitol S), three organic acids (malonic acid MO, citric acid C, malic acid MA) and two sugars (glucose Glu, fructose Fru). All NADES preparations remained stable for several weeks, consisted of components widely considered non-toxic for humans and were tested in different molar ratios of HBA and HBD, given in parentheses, with 20% m/m water addition. Figure 3 shows results for all investigated solvents in µg of spilanthol per ml of extraction agent. Figure 4 gives results grouped by HBD type used in NADES.

Interpreting results as given in Figure 3, polyalcohol 1,2-propanediol containing NADES ChCl/P (1:2) and (1:1) (244.58 and 221.44 µg/mL, *p* < 0.001) showed to be best suited for spilanthol extraction among all tested combinations, with ChCl/P (1:2) having no significant difference in yield to ethanol (245.93 µg/mL, *p* = 1). Some examples from literature show that NADES consisting of choline chloride and 1,2-propanediol could already be used successfully for the extraction of natural products from different sources, including hydrophilic and hydrophobic compounds from *Salvia miltiorrhiza* Bunge roots [39], α-mangostin from *Garcinia mangostana* L. pericarp [40], phenolics from *Salvia rosmarinus* Spenn. leaves [41], and flavonoids from citrus peel [42]. Other NADES were significantly less effective (*p* < 0.001), led by amide methylurea or malonic acid containing NADES ChCl/MeU (1:2), ChCl/MO (1:2) and ChCl/MeU (1:1) (208.12, 201.73, 190.28 µg/mL). The difference between ChCl/MeU (1:2) and ChCl/MO (1:2) was not significant (*p* = 0.854). The combination of choline chloride and methyl urea or malonic acid previously showed good results for the extraction of saponins from *Dioscorea nipponica* Makino rhizomes [43], ChCl/MeU was also used for the extraction of caffeoyl quinic acids from spent coffee grounds and flavonoids from citrus peel [42,44]. ChCl/MO already proved suitable or the extraction of curcuminoids from *Curcuma longa* L. [45]. Several other NADES with acids as HBD followed in the midfield for spilanthol extraction (180.85, 179.63, 150.51, 149.64, 107.69 µg/mL) before combinations containing polyalcohol glycerol (91.85, 88.06 µg/mL) and amide urea (86.13, 71.78 µg/mL). Lowest yields were achieved with polyalcohol sorbitol (34.44, 26.71 µg/mL) and sugars glucose (29.16, 20.84 µg/mL) and fructose (18.93, 16.72 µg/mL). The difference between NADES with 1,2-propanediol as best extraction agents and NADES with glucose or fructose performing poorly, could to some extent be attributed to a difference in viscosities, as reported for similar combinations with lower water content, and therefore lead to difficulty in mass transfer during extractions. However, the high viscosity of NADES consisting of choline chloride and glucose has been shown to decrease almost to levels of water when it is added in +20 to 25% (*v*/*v*) [46]. Viscosities can only play a partial role in results from a screening setup with water addition to NADES.

Within HBD groups as given in Figure 4, strong differences could be seen between tested polyalcohols (1,2-propanediol > glycerol > sorbitol, *p* < 0.001) and amides (methylurea > urea, *p* < 0.001). For acids, differences were less pronounced and a higher ratio of HBD to HBA seemed to be beneficial for yield. Taking this into account, an overall ranking in the efficiency of acid HBDs as malonic acid > citric acid > malic acid can be made, as data show ChCl/MO (1:2) > ChCl/C (1:2) > ChCl/MA (1:1) (*p* < 0.001) and ChCl/MO (1:1) > ChCl/C (1:1) > ChCl/MA (2:1) (*p* < 0.001). For sugars, glucose performed better than fructose in the sequence of ChCl/Glu (3:1) > ChCl/Glu (2:1) (*p* = 0.223) > ChCl/Fru (1:1) (*p* = 1) > ChCl/Fru (1:2) (*p* = 1). This internal ranking could be attributed to a higher ratio of HBA to sugar HBD having positive effects on spilanthol extraction, although yields were low and differences not significant.

Independent of NADES constituents used, future research will have to focus on the purification of spilanthol from NADES extracts. The recovery of non-protein bioactive compounds with aqueous two-phase systems has already been reviewed in detail and could be tested for spilanthol back-extraction from NADES [47]. Other techniques for the recovery of targets from NADES are liquid–liquid extractions with aprotic solvents, solid–liquid extractions through adsorption on resins, or precipitation by the addition of high amounts of antisolvents, like water [48].

### 2.3. Testing Effects on Spilanthol Extraction with NADES in Single-Factor Experimental Design

Two promising NADES in screening extractions of spilanthol were selected for further investigations. ChCl/P (1:2) proved to be equivalent to ethanol as an extraction solvent in screenings and was therefore first choice. ChCl/MeU (1:2) was chosen as the next best NADES in screenings, containing a different HBD. Although the advantage in extraction efficiency over ChCl/MO (1:2) was not significant, ChCl/MeU (1:2) showed a lower standard deviation between extraction replicates and was therefore deemed more adequate. The effect of time, temperature and water addition on spilanthol extraction was tested in a single-factor experimental design.

#### 2.3.1. Effect of Extraction Time

**Figure 5:**
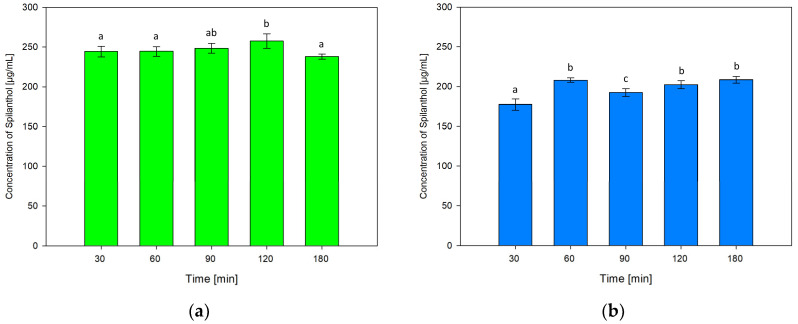
Concentration of spilanthol in NADES extracts with solvents ChCl/P (1:2) (**a**) and ChCl/MeU (1:2) (**b**) at various extraction times; data are given as means ± SD and different letters indicate significant differences between extraction conditions (*p* < 0.05).

For testing the influence of extraction time on the concentration of spilanthol in NADES extractions, durations of 30, 90, 120 and 180 min were investigated in addition to screening conditions of 60 min. Results are shown in Figure 5a for NADES ChCl/P (1:2) and Figure 5b for ChCl/MeU (1:2).

Extraction time had no significant effect on the performance of NADES ChCl/P (1:2) when comparing 30, 60 and 90 min. 30 min (244.44 µg/mL) and 60 min (244.58 µg/mL, *p* = 1) were almost equal, yield rose at 90 min (248.39 µg/mL, *p* = 0.884). Maximum extraction was reached at 120 min (257.52 µg/mL), which was significantly better than the 60 min screening condition (*p* = 0.032). The lowest spilanthol concentration was measured at 180 min (238.06 µg/mL), with no significant difference to screening (*p* = 0.522).

For ChCl/MeU (1:2), 30 min extraction time gave the lowest spilanthol concentration (177.32 µg/mL, *p* < 0.001). Screening conditions of 60 min (208.12 µg/mL) had no significant difference to 120 min (202.42 µg/mL, *p* = 0.388) and the maximum yield at 180 min (208.60 µg/mL, *p* = 1). A drop in spilanthol extraction was detected at 90 min (192.47 µg/mL), which ranged significantly below 60 min screening (*p* < 0.001).

Overall, the effect of extraction time on spilanthol extraction was low for both investigated NADES.

#### 2.3.2. Effect of Water Addition

**Figure 6:**
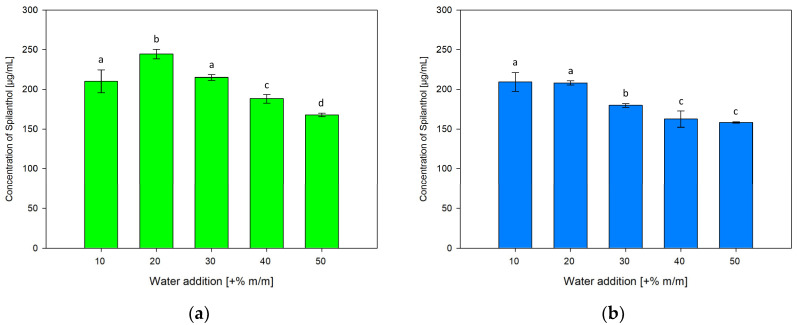
Concentration of spilanthol in NADES extracts with solvents ChCl/P (1:2) (**a**) and ChCl/MeU (1:2) (**b**) with varying water additions (+% m/m); data are given as means ± SD and different letters indicate significant differences between extraction conditions (*p* < 0.05).

To investigate the influence of water addition to NADES on spilanthol extraction effiency, ChCl/P (1:2) and ChCl/MeU (1:2) were individually prepared with 10, 30, 40 and 50% m/m water addition, differing from +20% m/m water used in NADES for screening extractions. Results are given in Figure 6a for ChCl/P (1:2) and Figure 6b for ChCl/MeU (1:2)

Both NADES showed a continuous reduction in spilanthol extraction with water additions over 20%. For ChCl/P (1:2), a rise from 10% (210.13 µg/mL) to significantly highest spilanthol concentration at 20% water in NADES (244.58 µg/mL, *p* < 0.001) could be detected, before reductions at 30% (215.07 µg/mL), 40% (188.14 µg/mL) and lowest concentration at 50% (167.68 µg/mL).

With ChCl/MeU (1:2), 10% (209.30 µg/mL) and 20% water addition (208.12 µg/mL) were highest in spilanthol yield and almost equal with no significant difference (*p* = 0.999). They both performed significantly better (*p* < 0.001) than 30% (179.88 µg/mL), 40% (162.46 µg/mL) and 50% (158.05 µg/mL).

Water addition has relevant effects on the H-bonding interaction between NADES components, with interactions weakening up to around +50% water before disappearing completely [49,50]. However, smaller amounts of water reduce the viscosity of NADES and influence polarity, which can be beneficial for the extraction of target compounds. Water addition can increase the extraction of hydrophilic targets, while reducing extraction of hydrophobic compounds [39]. It has been reported that the extraction of phenolics from rosemary leaves benefits from 30% water addition compared to pure NADES ChCl/P (1:2) [41]. For spilanthol with amphiphilic properties, beneficial effects on the extraction of up to 20% water addition can be seen in terms of yield and reproducibility, in particular with NADES ChCl/P (1:2).

#### 2.3.3. Effect of Extraction Temperature

**Figure 7:**
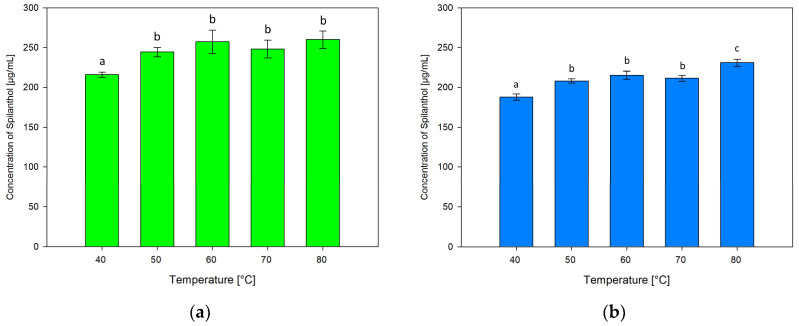
Concentration of spilanthol in NADES extracts with solvents ChCl/P (1:2) (**a**) and ChCl/MeU (1:2) (**b**) at various extraction temperatures; data are given as means ± SD and different letters indicate significant differences between extraction conditions (*p* < 0.05).

The effect of varying extraction temperatures of 40, 60, 70 and 80 °C in addition to 50 °C in screening extractions was tested. Figure 7 shows results for ChCl/P (1:2) (Figure 7a) and ChCl/MeU (1:2) (Figure 7b).

Extraction temperatures had similar effects on both NADES, with the lowest spilanthol extraction at 40 °C and the highest at 80 °C. For ChCl/P (1:2), 40 °C (216.17 µg/mL) performed significantly below other temperatures (*p* ≤ 0.001). 50 °C (244.58 µg/mL), 60 °C (257.22 µg/mL), 70 °C (248.24 µg/mL), and the highest concentration at 80 °C (260.21 µg/mL) had no significant differences (*p* = 0.133–0.989), although a slightly fluctuating plateau in spilanthol extraction seems to be established at temperatures of 60 °C and higher.

ChCl/MeU (1:2) follows a similar trend with significantly lowest extraction at 40 °C (188.01 µg/mL, *p* < 0.001), followed by 50 °C (208.12 µg/mL), 60 °C (215.39 µg/mL) and 70 °C (211.54 µg/mL) with non-significant differences (*p* = 0.063–0.676). Extraction then rises to a significant maximum at 80 °C (231.03 µg/mL, *p* < 0.001).

A rise in extraction efficiency with increasing temperature could be attributed to the reported decrease in density and viscosity for NADES [51]. The positive effect on natural product extraction with NADES has been shown before and could be the result of increased diffusion and solubility rates. At higher temperatures, plateauing or decreasing of yield can occur due to heat-sensitive targets or NADES components [41,42].

### 2.4. Validation of Quantitative Analyses

Linearity of measurements was confirmed for reference substance spilanthol in a working range of 10–500 μg/mL, showing a coefficient of determination of (*R*^2^) = 0.9995. Inter-day precision gave relative standard deviations (RSD) of 0.43% RSD for spilanthol at 10 µg/mL, 0.75% at 100 µg/mL, and 0.10% at 500 µg/mL. Intra-day precision showed RSDs of 1.44%, 0.11% and 0.12% at the same respective concentrations. Recovery rates at 10, 100 and 500 µg/mL spike concentrations were 94.57, 105.94 and 89.34%, respectively.

## 3. Materials and Methods

### 3.1. Plant Material

*Acmella oleracea* (L.) R.K.Jansen plants were raised from batches of seeds (Rühlemann, Horstedt, Germany) before separation into individual pots with garden soil and cultivation in a greenhouse at the Botanical Garden, University of Graz, over the months of May to September of 2019. Flower heads were continually harvested from July to September and dried at room temperature before storage in brown glass containers until further use. For extractions, the plant material was ground in a Retsch ZM100 centrifugal mill with 0.5 mm mesh (Retsch, Haan, Germany). Voucher specimens are deposited at the Department of Pharmacognosy, University of Graz.

### 3.2. Chemicals and Solvents

For NADES preparation, chemicals choline chloride, 1,2-propanediol, DL-malic acid, malonic acid, D-sorbitol, urea (all Thermo Fisher Scientific, Waltham, MA, USA), citric acid monohydrate, glycerol, D(−)-fructose (all Carl Roth, Karlsruhe, Germany), D(+)-glucose (Merck, Darmstadt, Germany) and methylurea (Acros Organics, Geel, Belgium) were used. Methylurea had a specified purity of 97%, and all other reagents were at least 98% or higher. In HPLC mobile phases, ultrapure water prepared from deionized water with a Barnstead MicroPure system (Thermo Fisher Scientific, Waltham, MA, USA), HPLC-grade acetonitrile (VWR, Radnor, PA, USA) and formic acid (Honeywell, Charlotte, NC, USA) were used. The solvent for NMR analyses was chloroform-d_1_ (VWR). Other solvents used in this study were partly denatured ethanol (96% *v*/*v*, AustrAlco, Spillern, Austria) methanol (≥99.9%, Carl Roth) and dichloromethane (≥99.5%, Carl Roth). Reference substance spilanthol was isolated in the course of this work with a purity of 97% as determined by NMR.

### 3.3. Preparation and Synthesis of NADES

A series of NADES based on cholin chloride as hydrogen bond acceptor (HBA) and varying hydrogen bond donors (HBD) was synthesized for use as extraction solvents in screening and single-factor design effect testing experiments, as listed in Table 2. A preparation technique based on heating–stirring approaches was developed, using an ultrasonic bath and incubator, enabling multiple parallel syntheses. NADES components were weighed into Erlenmeyer flasks according to the desired molar ratio. Water equaling +20% m/m was added to the mixture to reduce viscosity and therefore facilitate handling of the solvent. For single-factor design effect testing experiments, NADES with other percentages of water addition were prepared individually (10%, 30%, 40%, 50%). After sealing with glass stoppers and clamps, the mixtures were heated in a Memmert Ulm 500 incubator (Memmert, Schwabach, Germany) for 10 min and then treated in a Sonorex Super RK 255H ultrasonic bath (Bandelin electronic, Berlin, Germany) for 1 min. Cycles were repeated until homogenous, clear liquids were formed. For NADES containing sugars as HBD, an incubation temperature of 80 °C was necessary, all others were prepared at 50 °C. Solvents remained stable for several weeks, and no loss of water content could be detected, as checked gravimetrically.

### 3.4. Extraction and Isolation of Constituents

Two separate extractions of *Acmella oleracea* flower heads following the same procedure were undertaken as follows. 10 g of ground plant material was extracted three consecutive times with 150 mL of dichloromethane in an ultrasonic bath at room temperature for 10 min. The extract solutions were filtered through a paper filter, combined, and evaporated to dryness using a rotary evaporator. This yielded 1.16 g and 1.13 g of dry dichloromethane extract, respectively, which were individually fractioned by solid phase extractions on ISOLUTE C18(EC) columns (Biotage, Uppsala, Sweden) with 10 g of stationary phase. For both separations, columns were activated with 100 mL of acetonitrile, before equilibration with 100 mL of 35% acetonitrile in water. The extracts were dissolved in 140 mL of 35% acetonitrile and loaded onto the columns. Elutions with 100 mL of 35%, 58% and 100% acetonitrile led to three fractions each, of which fractions 2 (58% acetonitrile), both clear, pale-yellow solutions, showed the highest intensity of alkylamides without hydrophilic adulterants. They were combined and evaporated to dryness on a rotary evaporator to yield 164.60 mg. 80 mg of this sample were dissolved in 50% acetonitrile at a concentration of 10 mg/mL and used for isolation of main compounds with semipreparative HPLC on a Shimadzu system consisting of DGU-20A5R degassing unit, LC-20AT solvent delivery pump, SIL-10AF autosampler, CBM-20A controller, CTO-20AC column oven, SPD-M20A diode array detector and FRC-10A fraction collector (all Shimadzu, Kyoto, Japan). A Luna C-10(2) column with 250 × 10 mm and 10 µm particle size (Phenomenex, Torrance, CA, USA) served as the stationary phase, while the mobile phase consisted of water (A) and acetonitrile (B). Isocratic elution at 56% B was undertaken at a flow rate of 4 mL/min and 35 °C column temperature. Injection volume per HPLC run was 200 µL. This led to the isolation of alkylamides **3** (1.85 mg), **5** (26.26 mg) and **9** (2.68 mg) confirmed by NMR and GC-MS (Supplementary Materials Tables S1–S3, Figures S1–S3 and S5–S7), as well as ketoester **A** (acmellonate, 0.71 mg) confirmed by NMR (Supplementary Materials Table S4, Figure S4).

### 3.5. Extraction Protocol for NADES and Ethanol

For extractions with NADES and ethanol, a fast and easy extraction protocol was employed, building on descriptions by Gonzalez et al. [52]. In each extraction procedure, 50 mg of ground *Acmella oleracea* flower heads were brought into a 2 mL reaction tube before adding 1 mL of NADES or ethanol. Components were mixed with a plastic spatula for 1 min before extraction in an incubator. For screening experiments, extraction lasted for 60 min at 50 °C. A small plug of cotton wool to hold back plant material was added before 10 min centrifugation at 13,000 rpm in a Biofuge pico centrifuge (Heraeus, Hanau, Germany). An aliquot of 300 µL of clear supernatant was diluted with 1.2 mL of methanol (1:5 dilution) and centrifuged again for 10 min before HPLC analysis.

For testing effects of temperature and time on spilanthol extraction, additional temperatures of 40, 60, 70 and 80 °C and extraction times of 30, 90, 120 and 180 min were employed in separate extractions. All extractions were conducted in triplicate.

### 3.6. Chromatographic Techniques

#### 3.6.1. UHPLC-DAD-ESI-MS^n^ for Qualitative Analysis

A Dionex UltiMate 3000 RS system including a DAD detector, coupled to an LTQ XL linear ion-trap mass spectrometer with ESI ion source (all Thermo Fisher Scientific, Waltham, MA, USA) was used for initial qualitative analysis of a dichloromethane extract. The stationary phase was a Zorbax Eclipse Plus C18 column, 2.1 × 100 mm with 1.8 µm particle size (Agilent, Santa Clara, CA, USA) and the mobile phase consisted of water +0.1% formic acid (A) and acetonitrile (B). Gradient elution was performed at a flow rate of 0.25 mL/min, starting at 10% B, rising to 90% B at 20.0 min, followed by a fast rise to 100% B at 20.5 min, a plateau of 100% B until 23.0 min, a drop back to 10% B at 23.5 min, and re-equilibration until 30 min. Column temperature was 35 °C. An injection volume of 3 µL was used for dry dichloromethane extract dissolved in 50% acetonitrile (5 mg/mL). DAD-UV spectra in a wavelength range of 190 to 700 nm and mass spectral detection of *m*/*z* 50 to 2000 were performed. For MS detection, source heater temperature was 250 °C, sheath gas flow 27 arb (arbitrary units), auxiliary gas flow 8 arb and source voltage 4.2 kV in ESI positive mode.

#### 3.6.2. HPLC-DAD for Quantitative Analysis

All quantitative HPLC analyses were performed on a Vanquish Core System (Thermo Fisher Scientific), consisting of a VC-P20-A quaternary pump, VC-A12-A autosampler, VC-C10-A column compartment and VC-D11-A diode array detector. The stationary and mobile phases were identical to measurements for qualitative analysis. Elution also followed the same gradient, but with re-equilibration until 33 min. Column temperature was 35 °C, flow rate 0.25 mL/min and injection volume 5 µL for sample dilutions and 1 to 5 µL of serial dilutions (1:10, 1:100) prepared from spilanthol stock solution in methanol (1 mg/mL) to achieve desired reference concentrations. 2 and 10 µL injections were needed for recovery experiments. Measurements were performed in duplicate for samples and triplicate for validation. DAD-UV detection was recorded in a wavelength range of 200 to 400 nm, quantification wavelength for spilanthol was 230 nm.

#### 3.6.3. GC-MS Analysis

GC-MS measurements of isolated substances were performed on a 7890A GC system with 5975C VL mass selective detector (Agilent, Santa Clara, CA, USA). The capillary column was an HP-5ms, 30 m × 0.25 mm, 0.25 µm film thickness, with (5%-phenyl)-methylpolysiloxane phase (Agilent). Mobile phase was helium 5.6 at 1 mL/min flow rate. The temperature program started at 60 °C with 1 min hold time, then rising at +6 °C/min to 280 °C and 10 min hold. Injection volume was 1 µL with 30:1 split, at an injection temperature of 260 °C. EI-MS detection was performed at 70 eV, interface temperature 280 °C, ion source temperature 230 °C, quadrupole temperature 150 °C, with scan parameters 40–600 amu. Identification through comparison of MS data with reference spectra was undertaken using databases NIST 17 and Wiley 138.

### 3.7. NMR Analysis

Isolated substances were measured on a Bruker Advance II NMR-spectrometer (Bruker, Rheinstetten, Germany) at 700 MHz proton frequency and 25 °C in chloroform-d_1_ with an internal standard of TMS or on a Bruker Neo spectrometer at 400 MHz proton frequency. Data sets comprised ^1^H, ^13^C and 2D experiments COSY, HSQC and HMBC.

### 3.8. Validation of Quantitative Analyses

To confirm linearity of HPLC quantifications within the working range, spilanthol concentrations 10, 30, 50, 100, 300, and 500 µg/mL were analyzed by 1, 3 and 5 µL injections of serial dilutions (1:10, 1:100) prepared from spilanthol stock solution in methanol (1 mg/mL). For determination of intra-day precision, the same spilanthol dilutions (1:10, 1:100) were injected six consecutive times in volumes of 1 or 5 µL to give target concentrations of 10, 100 and 500 µg/mL. For inter-day precision, those samples were injected 3 times on two consecutive days. Recovery rates were determined by spiking an ethanolic extract of *Acmella oleracea* flower heads with spilanthol solutions to reach spike concentrations of 10, 100 and 500 µg/mL.

### 3.9. Statistical Analysis

Statistical analyses were performed in IBM SPSS Statistics 29. A one-way analysis of variance (ANOVA) with post-hoc *t*-test was used for mean comparisons, with the α-level set to 0.05.

## 4. Conclusions

In this work, the screening of choline chloride-based NADES and ethanol as solvents for the green extraction of spilanthol from *Acmella oleracea* flower heads showed the best results for choline chloride with 1,2-propanediol (1:2 molar ratio, 20% water addition), comparable to yields achieved with ethanol. Methylurea and malonic acid also showed promising results as HBD in combination with choline chloride. In further experiments with NADES ChCl/P (1:2) and ChCl/MeU (1:2), extraction time had the least effect on spilanthol extraction with NADES, while yield fell with water addition over 20% and increased with extraction temperature up to 80 °C. The great potential of NADES as green extraction agents for natural products could be emphasized. As an alternative to classic organic solvents, NADES can lead to spilanthol rich extracts from *Acmella oleracea* plant material, suitable for medicinal purposes.

## Figures and Tables

**Table 1:** Qualitative analysis of *Acmella oleracea* dichloromethane extract.

Nr.	RT [min]	[M + H]^+^	MS^n^ [*m*/*z*], Relative Intensity (%) ^1^	Mol. Mass	Tentative Identification
**1**	11.97	204	MS^2^ [204]: 176 (19), **148** (100), **131** (11), 120 (11), **105** (73), **103** (10)MS^3^ [148]: **131** (11), 120 (37), 106 (31), **105** (100), **103** (61), 79 (61)	203	(2Z)-N-isobutyl-2-nonene-6,8-diynamide [3,4,5,6]
**2**	12.85	230	MS^2^ [230]: **174** (43), **157** (16), **131** (100), **129** (54), 116 (19), 91 (49)MS^3^ [131]: 116 (27), 91 (100)	229	(2E,4Z)-N-isobutyl-2,4-undecadiene-8,10-diynamide [3,4,6]
**3**	13.29	232	MS^2^ [232]: **176** (100), **159** (23), **133** (89), **131** (20), 105 (70), 91 (28)MS^3^ [176]: **159** (100), 158 (40), 149 (59), 148 (74), 135 (97), 134 (40), **133** (55), **131** (96), 121 (32), 117 (32), 107 (22), 105 (59), 93 (17), 91 (40)	231	(2E)-N-isobutyl-2-undecene-8,10-diynamide ^2^ [3,4,5,6]
**4**	13.87	222	MS^n^ identical to spilanthol	221	Spilanthol isomer [5]
**5**	14.00	222	MS^2^ [222]: **123** (100), 81 (60)MS^3^ [123]: 81 (100), 67 (17)	221	(2E,6Z,8E)-N-isobutyl-2,6,8-decatrienamide (spilanthol) ^2^ [3,4,5,6]
**6**	14.19	222	MS^n^ identical to spilanthol	221	Spilanthol isomer [5]
**7**	14.39	246	MS^2^ [246]: **176** (100), **159** (19), **133** (71), **131** (16), 105 (54), 91 (26)MS^3^ [176]: **159** (99), 158 (41), 149 (48), 148 (75), 135 (74), 134 (33), **133** (66), **131** (100), 121 (24), 117 (29), 107 (19), 105 (82), 93 (13), 91 (49)	245	(2E)-N-(2-methylbutyl)-2-undecene-8,10-diynamide [3,4,5,6]
**8**	14.61	258	MS^2^ [258]: 230 (13), **202** (100), **185** (24), 174 (10), 161 (14), **159** (28), **157** (37), 143 (14), 133 (10), 131 (43), 129 (22), 117 (47), 105 (10), 91 (10)MS^3^ [202]: **185** (65), 184 (43), 175 (64), 174 (100), 167 (30), 161 (81), 160 (66), **159** (22), **157** (76), 148 (14), 146 (24), 143 (41), 136 (11), 133 (63), 131 (33), 129 (23), 119 (17), 117 (21)	257	(2E,7Z)-N-isobutyl-2,7-tridecadiene-10,12-diynamide [3,4,5,6]
**9**	15.17	236	MS^2^ [236]: **123** (100), 81 (61)MS^3^ [123]: 81 (100), 67 (18)	235	(2E,6Z,8E)-N-(2-methylbutyl)-2,6,8-decatrienamide (homospilanthol) ^2^ [3,4,5,6]
**10**	15.55	220	MS^2^ [220]: 202 (29), 192 (11), **164** (30), 149 (11), **147** (52), **121** (100), **119** (22), 93 (23)MS^3^ [121]: 93 (100), 79 (28)	219	UnidentifiedIsobutylamide [4]
**11**	15.75	248	MS^2^ [248]: **175** (23), **149** (100), **147** (21), 142 (47), 107 (11)MS^3^ [149]: 121 (95), 107 (100), 93 (90), 81 (41), 67 (12)	247	(2E,4E,8Z,10E)-N-isobutyl-dodeca-2,4,8,10-tetraenamide [3,4,5]

^1^ Characteristic fragment ions associated with the amide group are given in bold. ^2^ Identification confirmed by NMR after isolation.

**Table 2:** NADES preparations used as extraction agents in screening and single-factor design effect testing experiments.

Abbreviation	HBA	HBD	Molar Ratio	Water (+% m/m) ^1^
ChCl/P (1:1)	Choline chloride	1,2-Propanediol	1:1	20
ChCl/P (1:2)	Choline chloride	1,2-Propanediol	1:2	10, 20, 30, 40, 50
ChCl/Gly (1:1)	Choline chloride	Glycerol	1:1	20
ChCl/Gly (1:2)	Choline chloride	Glycerol	1:2	20
ChCl/S (2:1)	Choline chloride	Sorbitol	2:1	20
ChCl/S (3:1)	Choline chloride	Sorbitol	2:1	20
ChCl/MeU (1:1)	Choline chloride	Methylurea	1:1	20
ChCl/MeU (1:2)	Choline chloride	Methylurea	1:2	10, 20, 30, 40, 50
ChCl/U (1:1)	Choline chloride	Urea	1:1	20
ChCl/U (1:2)	Choline chloride	Urea	1:2	20
ChCl/MO (1:1)	Choline chloride	Malonic acid	1:1	20
ChCl/MO (1:2)	Choline chloride	Malonic acid	1:2	20
ChCl/C (1:1)	Choline chloride	Citric acid monohydrate	1:1	20
ChCl/C (1:2)	Choline chloride	Citric acid monohydrate	1:2	20
ChCl/MA (1:1)	Choline chloride	Malic acid	1:1	20
ChCl/MA (2:1)	Choline chloride	Malic acid	2:1	20
ChCl/Glu (2:1)	Choline chloride	Glucose	2:1	20
ChCl/Glu (3:1)	Choline chloride	Glucose	3:1	20
ChCl/Fru (1:1)	Choline chloride	Fructose	1:1	20
ChCl/Fru (1:2)	Choline chloride	Fructose	1:2	20

^1^ Water additions other than +20% m/m were prepared individually for effect testing experiments.

## Data Availability

Data are included in this article.

## Supplementary Materials

The following supporting information can be downloaded at: https://www.mdpi.com/article/10.3390/molecules29030612/s1, Figure S1: Structure of substance **3** (2E)-N-isobutyl-2-undecene-8,10-diynamide; Figure S2: Structure of substance **5** (2E,6Z,8E)-N-isobutyl-2,6,8-decatrienamide (spilanthol); Figure S3: Structure of substance **9** (2E,6Z,8E)-N-(2-methylbutyl)-2,6,8-decatrienamide (homospilanthol); Figure S4: Structure of substance **A** (7Z,9E)-(2-Oxoundeca-7,9-dien-1-yl) 3-methylbut-2-enoate (acmellonate); Figure S5: EI-MS spectrum of substance **3** (2E)-N-isobutyl-2-undecene-8,10-diynamide (**a**) with database reference spectrum (**b**); Figure S6: EI-MS spectrum of substance **5** (2E,6Z,8E)-N-isobutyl-2,6,8-decatrienamide (spilanthol) (**a**) with database reference spectrum (**b**); Figure S7: EI-MS spectrum of substance **9** (2E,6Z,8E)-N-(2-methylbutyl)-2,6,8-decatrienamide (homospilanthol) (**a**) with database reference spectrum (**b**); Table S1: NMR-data of substance **3** (2E)-N-isobutyl-2-undecene-8,10-diynamide in CDCl_3_ (700 MHz); Table S2: NMR-data of substance **5** (2E,6Z,8E)-N-isobutyl-2,6,8-decatrienamide (spilanthol) in CDCl_3_ (400 MHz); Table S3: NMR-data of substance **9** (2E,6Z,8E)-N-(2-methylbutyl)-2,6,8-decatrienamide (homospilanthol) in CDCl_3_ (700 MHz); Table S4: NMR-data of substance **A** (7Z,9E)-(2-Oxoundeca-7,9-dien-1-yl) 3-methylbut-2-enoate (acmellonate) in CDCl_3_ (700 MHz).
