# Supplementary material for: Natural Deep Eutectic Solvents for the Extraction of Spilanthol from Acmella oleracea (L.) R.K.Jansen
Source: Molecules. 2024 Jan 27;29(3):612. doi: 10.3390/molecules29030612 (PMC10856685; doi:10.3390/molecules29030612)
Supplement: Supplementary file 1 [file molecules-29-00612-s001.zip › molecules-2811545-supplementary.pdf]

Article

Natural Deep Eutectic Solvents for the Extraction of Spilanthol from *Acmella oleracea* (L.) R.K.Jansen

Supplementary Materials

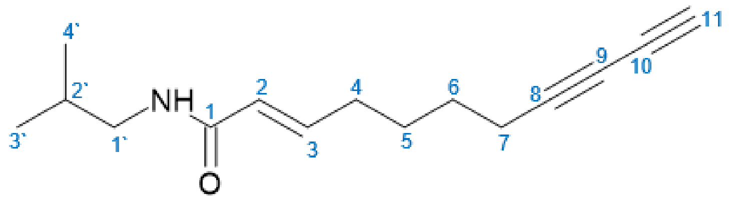

Figure S1. Structure of substance **3** (2E)-N-isobutyl-2-undecene-8,10-diynamide.

Table S1. NMR-data of substance **3** (2E)-N-isobutyl-2-undecene-8,10-diynamide in $CDCl_3$ (700 MHz).

Position	$\delta^{13}C$ [ppm]	$\delta^{1}H$ [ppm], Mult.[1] (J in Hz)
1	165.9	-
2	124.1	5.78, d (15.2)
3	143.8	6.82, dt (15.2, 7.0)
4	31.3	2.20, q (6.3)
5	27.2	1.58
6	27.4	1.58
7	18.9	2.28, m
8	77.9	-
9	65.0	-
10	68.4	-
11	64.7	1.97, s
1′	46.9	3.16, t (6.4)
2′	28.6	1.80, m
3′	20.2	0.93, d (6.5)
4′	20.2	0.93, d (6.5)
-NH	-	5.44, brs

[1] s: singlet, brs: broad singlet, d: doublet, t: triplet, dt: doublet of triplets, q: quadruplet, m: multiplet, multiplicities are given only for not overlapping resonances.

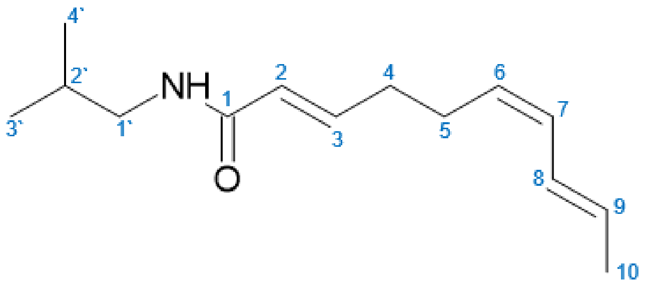

Figure S2. Structure of substance **5** (2E,6Z,8E)-N-isobutyl-2,6,8-decatrienamide (spilanthol).

Table S2. NMR-data of substance **5** (2E,6Z,8E)-N-isobutyl-2,6,8-decatrienamide (spilanthol) in $CDCl_3$ (400 MHz).

Position	δ13C [ppm]	δ1H [ppm], Mult.[1] (J in Hz)
1	166.0	-
2	124.2	5.81, brd (15.4)
3	143.5	6.83, dt (15.3, 6.7)
4	32.1	2.26, m
5	26.4	2.33, m
6	127.7	5.26, dt (10.7, 6.9)
7	129.4	5.98, t (10.8)
8	126.7	6.29, dd (13.0, 11.0)
9	130.0	5.69
10	18.3	1.78, d (6.5)
1′	46.9	3.14, t (6.6)
2′	28.6	1.80, m
3′	20.1	0.92, d (6.7)
4′	20.1	0.92, d (6.7)
-NH	-	5.68

[1] d: doublet, brd: broad doublet, dd: doublet of doublets, t: triplet, dt: doublet of triplets, m: multiplet, multiplicities are given only for not overlapping resonances.

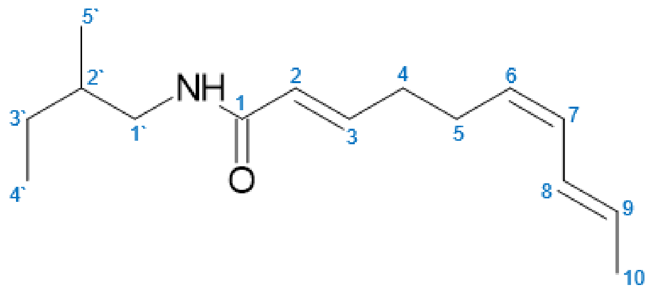

Figure S3. Structure of substance **9** (2E,6Z,8E)-N-(2-methylbutyl)-2,6,8-decatrienamide (homospilanthol).

Table S3. NMR-data of substance **9** (2E,6Z,8E)-N-(2-methylbutyl)-2,6,8-decatrienamide (homospilanthol) in $CDCl_3$ (700 MHz).

Position	δ¹³C [ppm]	δ¹H [ppm], Mult.[1] (J in Hz)
1	166.0	-
2	124.1	5.78, d (15.3)
3	143.5	6.82, td (6.7, 15.2)
4	32.2	2.26, q (7.4)
5	26.4	2.32, q (7.4)
6	127.7	5.27, dt (11.0, 7.5)
7	129.4	5.97, t (11.0)
8	126.7	6.29, t (13.0)
9	130.0	5.71, m
10	18.3	1.78, d (6.7)
1′	45.1	3.26, m
		3.14, m
2′	35.0	1.58
3′	27.0	1.41, m
		1.16, m
4′	11.3	0.91
5′	17.2	0.91
-NH	-	5.41, brs

[1] brs: broad singlet, d: doublet, td: triplet of doublets, t: triplet, dt: doublet of triplets, q: quadruplet, m: multiplet, multiplicities are given only for not overlapping resonances.

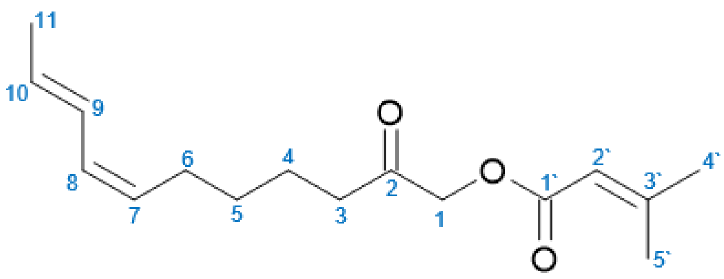

Figure S4. Structure of substance **A** (7Z,9E)-(2-Oxoundeca-7,9-dien-1-yl) 3-methylbut-2-enoate (acmellonate).

Table S4. NMR-data of substance **A** (7Z,9E)-(2-Oxoundeca-7,9-dien-1-yl) 3-methylbut-2-enoate (acmellonate) in $CDCl_3$ (700 MHz).

Position	$\delta^{13}C$ [ppm]	$\delta^{1}H$ [ppm], Mult.[1] (J in Hz)
1	67.4	4.64, s
2	204.7	-
3	38.7	2.44, t (7.2)
4	22.9	1.64, quint (7.6)
5	29.2	1.40, quint (7.6)
6	27.3	2.17
7	128.8	5.25, dd (10.8, 7.6)
8	129.0	5.95, t (11.4)
9	126.9	6.29, t (13.1)
10	129.4	5.67, m
11	18.3	1.77, d (6.6)
1′	165.6	-
2′	114.8	5.80, s
3′	159.0	-
4′	27.5	1.93, s
5′	20.4	2.18, s

[1] s: singlet, d: doublet, dd: doublet of doublets, t: triplet, quint: quintet, m: multiplet, multiplicities are given only for not overlapping resonances.

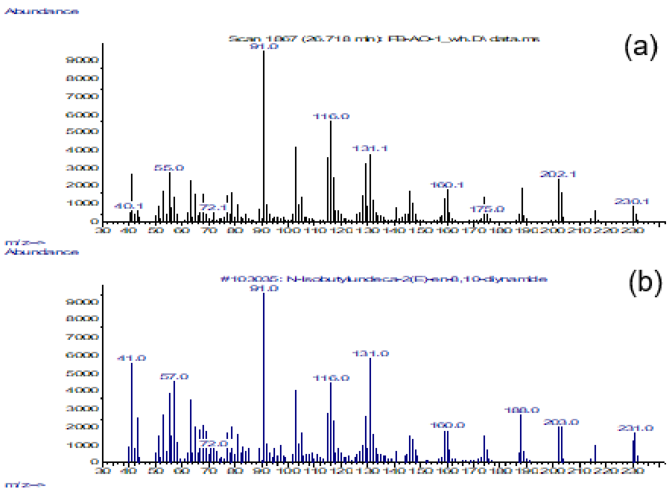

Figure S5. EI-MS spectrum of substance **3** (2E)-N-isobutyl-2-undecene-8,10-diynamide (**a**) with database reference spectrum (**b**).

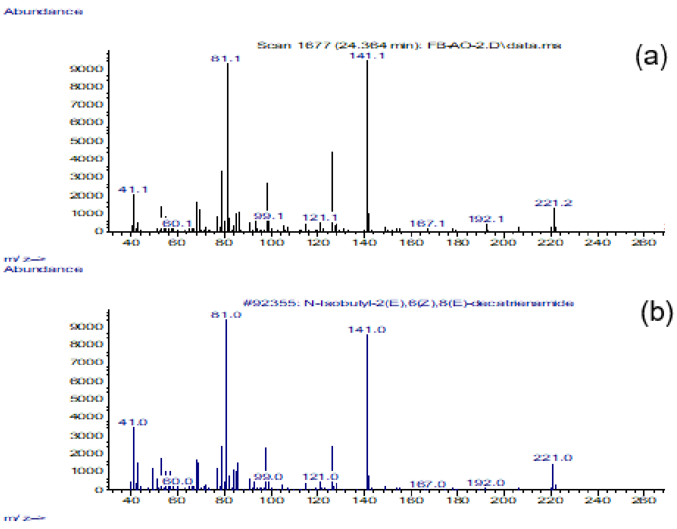

Figure S6. EI-MS spectrum of substance **5** (2E,6Z,8E)-N-isobutyl-2,6,8-decatrienamide (spilanthol) (**a**) with database reference spectrum (**b**).

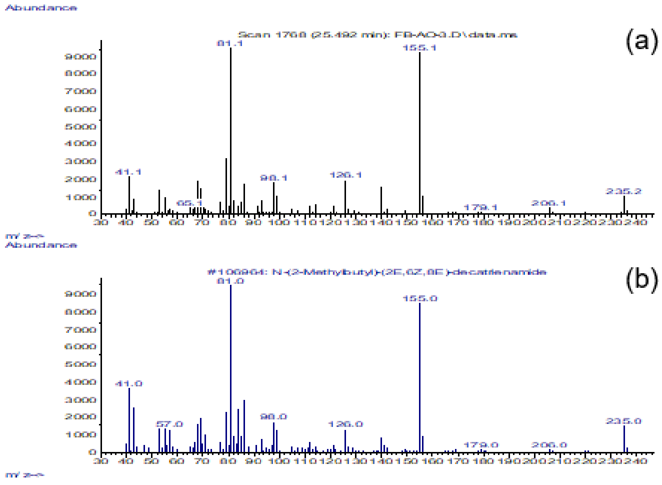

Figure S7. EI-MS spectrum of substance **9** (2E,6Z,8E)-N-(2-methylbutyl)-2,6,8-decatrienamide (homospilanthol) (**a**) with database reference spectrum (**b**).
